# Oral vaccination with recombinant *Lactobacillus plantarum* encoding *Trichinella spiralis* inorganic pyrophosphatase elicited a protective immunity in BALB/c mice

**DOI:** 10.1371/journal.pntd.0009865

**Published:** 2021-10-26

**Authors:** Chen Xi Hu, Yang Xiu Yue Xu, Hui Nan Hao, Ruo Dan Liu, Peng Jiang, Shao Rong Long, Zhong Quan Wang, Jing Cui

**Affiliations:** Department of Parasitology, Medical College, Zhengzhou University, Zhengzhou, PR China; US Food and Drug Administration, UNITED STATES

## Abstract

**Background:**

Trichinellosis is a serious zoonotic disease distributed around the world. It is needed to develop a safe, effective and feasible anti-*Trichinella* vaccine for prevention and control of trichinellosis. The aim of this study was to construct a recombinant *Lactobacillus plantarum* encoding *Trichinella spiralis* inorganic pyrophosphatase (TsPPase) and investigate its immune protective effects against *T*. *spiralis* infection.

**Methodology/Principal findings:**

The growth of recombinant *L*. *plantarum* was not affected by TsPPase/pSIP409-pgsA′ plasmid, and the recombinant plasmid was inherited stably in bacteria. Western blot and immunofluorescence assay (IFA) indicated that the rTsPPase was expressed on the surface of recombinant *L*. *plantarum*. Oral vaccination with rTsPPase induced higher levels of specific serum IgG, IgG1, IgG2a and mucosal secretory IgA (sIgA) in BALB/c mice. ELISA analysis revealed that the levels of IFN-γ and IL-4 released from spleen, mesenteric lymph nodes and Peyer’s patches were evidently increased at 2–4 weeks following vaccination, compared to MRS (De Man, Rogosa, Sharpe) medium control group (*P* < 0.05). Immunization of mice with rTsPPase exhibited a 67.18, 54.78 and 51.91% reduction of intestinal infective larvae, adult worms and muscle larvae at 24 hours post infection (hpi), 6 days post infection (dpi) and 35 dpi, respectively (*P* < 0.05), and the larval molting and development was significantly inhibited by 45.45% at 24 hpi, compared to the MRS group.

**Conclusions:**

TsPPase plays a crucial role in *T*. *spiralis* molting and development, oral vaccination with rTsPPase induced a significant local mucosal sIgA response and systemic Th1/Th2 immune response, and immune protection against *T*. *spiralis* infection in BALB/c mice.

## Author summary

In the previous study, a *Trichinella spiralis* inorganic pyrophosphatase (TsPPase) was expressed and its role in larval molting and development was observed. In this study, a recombinant TsPPase/pSIP409-pgsA′ plasmid was constructed and transferred into *Lactobacillus plantarum* NC8, the rTsPPase was expressed on the surface of recombinant *L*. *plantarum* NC8. Oral immunization of mice with rTsPPase DNA vaccine elicited a high level of specific serum IgG, IgG1, IgG2a and mucosal secretory IgA (sIgA). The levels of IFN-γ and IL-4 released from spleen, mesenteric lymph nodes and Peyer’s patches were evidently increased at 2–4 weeks following vaccination. Immunization of mice with rTsPPase showed a significant reduction of intestinal infective larvae, adult worms and muscle larvae, and intestinal larval molting and development was significantly suppressed. The results indicated that oral vaccination with rTsPPase elicited a significant local mucosal sIgA response and specific systemic Th1/Th2 immune response, and an obvious protective immunity against *T*. *spiralis* infection.

## Introduction

Trichinellosis is mainly caused by the zoonotic nematode *Trichinella spiralis* which is a tissue-dwelling nematode infected over 150 kinds of mammals over the world [1,2]. Humans acquire trichinellosis by ingesting raw or semi-raw meat containing *Trichinella* larvae. Fifteen trichinellosis outbreaks were documented in China from 2004 to 2009, which consisted of 1387 cases and 4 deaths [3]. Trichinellosis has become a major food-borne zoonosis with economic, social and public health impacts in endemic countries [4–6]. However, it is difficult to eliminate animal *Trichinella* infection due to its broad animal hosts and no practicable anti-*Trichinella* vaccines. Hence, there is an urgent need to develop a safe, effective and feasible anti-*Trichinella* vaccine for control of *Trichinella* infection in food animals and for food safety.

The life cycle of *T*. *spiralis* is complex, including various stages: newborn larvae (NBL), muscle larvae (ML), intestinal infective larvae (IIL) and adult worm (AW). IIL develop into AW after 4 molts. Larval molting is the key step of *Trichinella* growth and development in host intestine. If the larval molting is inhibited, the larval growth and development were blocked, and the larvae cannot be developed to adulthood [7].

At present, there are some studies on immunoprophylaxis with recombinant *T*. *spiralis* proteins, such as TsCB, TsSerp and TsASP [8–10], mainly by subcutaneous vaccination. However, *Trichinella* infection was principally resulted from oral ingestion of contaminated infected meat, subcutaneous vaccination is not an appropriate immunization approach for anti-*Trichinella* vaccine [11,12].

In recent years, attenuated *Salmonella* strains have been used to construct anti-*Trichinella* vaccine, oral vaccination with attenuated *Salmonella* vaccine provided a partial immune protection against *Trichinella* infection [13–15], but the *Salmonella* was a kind of pathogenic bacteria, *Salmonella* infection causes enteric fever or diarrhea, often resulting in death of humans and animals [16]. The recombinant plasmid in attenuated *Salmonella* vaccine was unstable *in vivo*, and the level of exogenous gene expression was low, and the recombinant plasmid in attenuated *Salmonella* was sometimes lost following immunization [17].

Lactic acid bacteria (LAB) were a kind of Gram-positive bacteria, which was common in daily life. There were many foods containing LAB, it was mainly related to the probiotic effect of LAB and it had no endotoxin [18]. LAB included *Lactobacillus*, *Lactococcus* and *Bifidobacterium*, which widely distributed in human and animals [19]. LAB was mainly distributed in gastrointestinal tract, reproductive system, urinary system and mucous membrane, there lots of LAB proteins were expressed [20]. The proteins stimulate the mucosal reaction, effectively induce the immune response of host and produce the corresponding antibodies [21]. Therefore, LAB can be used as a good carrier for the construction of immune protective vaccine against *Trichinella* infection.

In our previous study, a *T*. *spiralis* inorganic pyrophosphatase (TsPPase) was cloned, expressed and characterized, the TsPPase was highly expressed in IIL stage during *T*. *spiralis* lifecycle, and played a crucial role in larval molting and development [22]. To further evaluate the immune protective effect of TsPPase, an anchored expression vector pSIP409-pgsA′ was selected in this study, the vector contains a pgsA’ protein anchoring sequence for attaching the gene encoding TsPPase, which was supposed to be expressed on the surface of a probiotic recombinant strain *Lactobacillus plantarum* [23]. Specific humoral and cellular immune responses and protective efficacy against *T*. *spiralis* challenge were observed by oral vaccination with recombinant *L*. *plantarum* expressing TsPPase in BALB/c mice.

## Materials and methods

### Ethics statement

Ethical approval was acquired from the Institutional Life Science Ethics Committee of Zhengzhou University (No. SCXK 2017–0001).

### Parasites, experimental animals and *L*. *plantarum* NC8

*T*. *spiralis* strain (ISS 534) used in this study was obtained from a domestic swine in central China [24]. The parasite passage was carried out in mice. Female BALB/c mice with six-week-old were purchased from Henan Provincial Experimental Animal Center (Zhengzhou, China). *L*. *plantarum* NC8 was provided by Professor Gui Liang Yang from College of Animal Science and Technology, Jilin Agricultural University, China [25].

### Preparation of anti-rTsPPase serum

The recombinant plasmid pQE-80L/TsPPase was constructed in our previous study [22]. The rTsPPase protein was induced in an *E*. *coli* BL21 expression system and purified by Ni-NTA-Sefinose resin (Sangon Biotech, Shanghai, China) [26], the purified rTsPPase was used to immunize BALB/c mice three times with an interval of two weeks, and anti-rTsPPase serum was obtained [27].

### Construction of recombinant *L*. *plantarum* NC8

The full-length cDNA sequence of TsPPase gene was amplified by PCR with specific primers carrying Xba I and Hind III, then cloned into pSIP409-pgsA′. The recombinant pSIP409-pgsA′/TsPPase was transferred into *L*. *plantarum* NC8 by electroporation. PCR was used to detect whether the pSIP409-pgsA′/TsPPase could be observed in each generation of recombinant bacteria, therefore to ascertain the genetic stability of the pSIP409-pgsA′/TsPPase. In order to evaluate the effect of pSIP409-pgsA′/TsPPase on the growth of *L*. *plantarum*, the recombinant *L*. *plantarum* NC8 was cultured in MRS broth culture medium at 30°C for 24 h, and OD_600nm_ of bacterium solution was measured by ultraviolet spectrophotometer every 2 hours during the cultivation, *L*. *plantarum* NC8 was used as control. In addition, to investigate the survival of recombinant *L*. *plantarum* NC8 under different pH conditions, gastric environment with different pH was simulated *in vitro*. Briefly, the recombinant *L*. *plantarum* NC8 was cultured in different pH for 4 h at 30°C, and then the bacterial solution was extracted and cultured in MRS medium per hour during the cultivation. The number of bacteria in MRS medium was counted and compared in different pH groups. The data were compared in the form of logarithms (log) due to the large number of bacteria [28].

### Western blot and immunofluorescence assay (IFA)

The recombinant *L*. *plantarum* NC8 was cultured in MRS medium supplemented 50 ng/mL peptide SppIP (sakacin P) and 10 μg/mL erythromycin till OD_600nm_ 0.3 at 30°C, then the bacterial solution was continuously cultured at the same condition for 8 h to induce the expression of TsPPase. Western blot was performed to detect the TsPPase expression in recombinant *L*. *plantarum* NC8 [29]. Soluble proteins of recombinant *L*. *plantarum* NC8 carrying pSIP409-pgsA′/TsPPase was transferred to nitrocellulose membranes (Merck Millipore, Billerica, MA, USA), and probed with anti-rTsPPase serum (1:100). Then the membranes were incubated with HRP-anti-mouse IgG conjugate (1:10000; Southern Biotech, Tuscaloosa, AL, USA), and finally colored with 3, 3’-diaminobenzidine tetrahydrochloride (DAB; Sigma-Aldrich, St. Louis, MO, USA). The *L*. *plantarum* NC8 without TsPPase gene was used as the negative control. Additionally, the IFA was also performed to detect the expression of TsPPase on the surface of recombinant *L*. *plantarum* NC8. After being blocked with 1% bovine serum albumin (BSA), the recombinant *L*. *plantarum* NC8 was incubated with anti-rTsPPase serum, infection serum or normal serum (1:100) at 37°C for 2 h, then incubated with cy3/FITC- anti-mouse IgG conjugate (1:100; Santa Cruz Biotech, Dallas, Texas, USA), finally examined with fluorescence microscopy (Olympus, Tokyo, Japan) [30].

### Immunization of BALB/c mice with recombinant *L*. *plantarum* NC8

**Fig 1:**
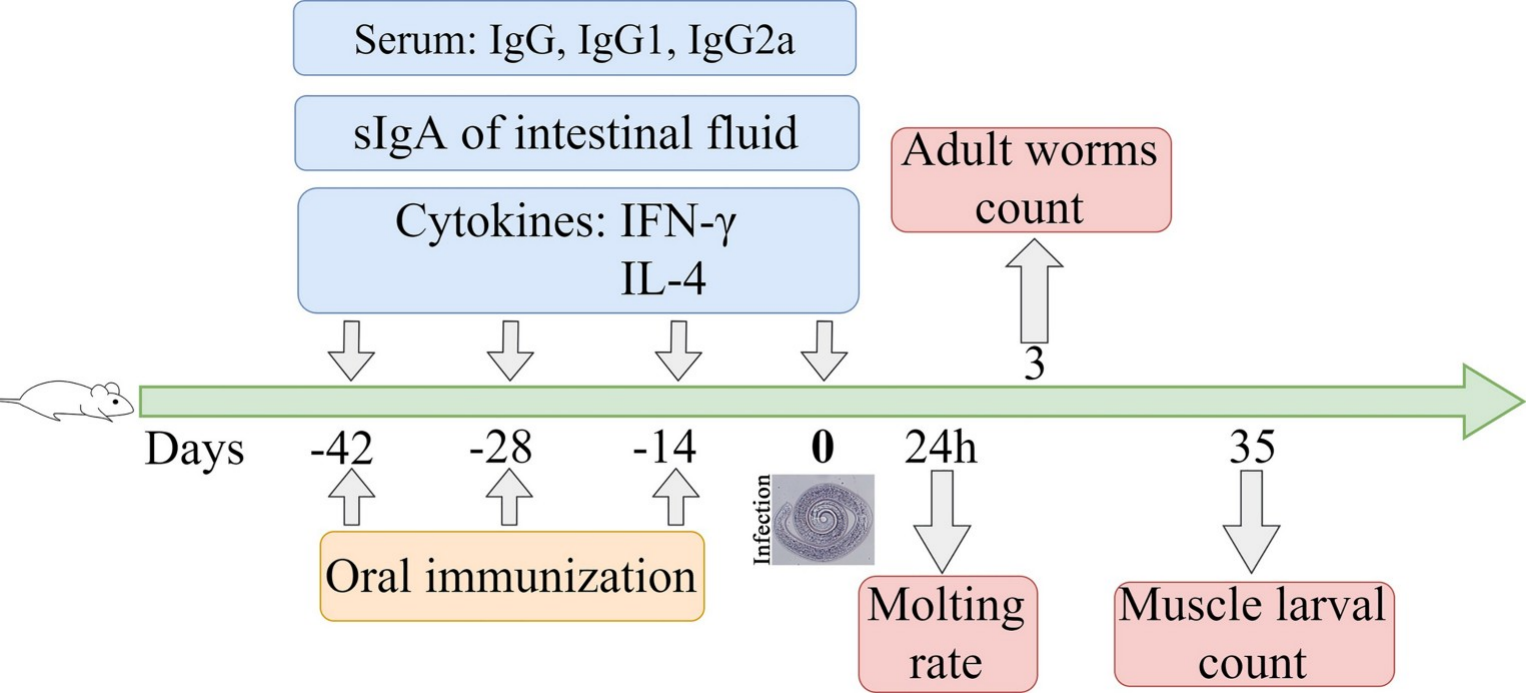
The designed immunization scheme and detection protocol. A total of 3 oral immunizations were performed, and 5 mice of each group were selected for sIgA and cytokine detection at 2 weeks after each immunization. Serum specific anti-TsPPase antibodies (total IgG, IgG1and IgG2a) were also measured by ELISA with *T*. *spiralis* ML excretory-secretory (ES) antigens 2 weeks following each immunization. After being challenged with *T*. *spiralis* larvae, the 24 h IIL, AW and ML were recovered at 24 hpi, 6 and 35 dpi to evaluate the immune protective efficacy of recombinant NC8 against *T*. *spiralis* challenge infection, and the larval molting rate of 24 h IIL was also detected.

One hundred and fifty female BALB/c mice were equally divided into three groups (50 animals per group): recombinant *L*. *plantarum* NC8 group, NC8 control group and MRS control group. Each group of mice was orally administrated with recombinant *L*. *plantarum* NC8, *L*. *plantarum* NC8 or MRS medium, respectively. The recombinant *L*. *plantarum* NC8 and NC8 control groups were inoculated with 200 μl bacterial solution for three consecutive days at each vaccination, the bacterial number was 1 × 10^10^ colony-forming unit (CFU)/ml, and the MRS group was given with 200 μl MRS medium. Two boost immunizations were administered as the same dosage of recombinant NC8 at a 14-day interval. Approximately 100 μl of blood samples was collected from each mouse tail prior to vaccination and at 2, 4 and 6 weeks after vaccination. Individual serum sample was stored at– 80°C until use. Five mice of each group were sacrificed at weeks 0, 2, 4 and 6 weeks after vaccination, and the intestine, spleens, mesenteric lymph nodes (MLNs) and Peyer’s patches (PPs) were collected, respectively. The scheme of vaccination protocol was shown in Fig 1.

### Measurement of serum anti-rTsPPase antibodies by ELSIA

Serum specific anti-rTsPPase IgG, IgG1 and IgG2a were detected by ELISA with ML excretory-secretory (ES) antigens [31,32]. In brief, 2.5 μg/ml ML ES was used to coat ELISA plate at 4°C overnight, then the plate was blocked with 5% skimmed milk in PBS-0.5% Tween (PBST) for 2 h at 37°C. After being washed using PBST, the plate was probed with anti-rTsPPase serum (1:100) for 1 h at 37°C, and then incubated with HRP-anti-mouse IgG conjugate (1:10000; Southern Biotech) at 37°C for 1 h. Following washes, o-phenylenediamine dihydrochloride (OPD; Sigma) was used as the substrate for coloration, the OD values at 492 nm were measured with a microplate reader (Tecan, Schweiz, Switzerland) [33,34].

### Assessment of intestinal total sIgA and TsPPase-specific sIgA

Total and TsPPase-specific secretory IgA (sIgA) in intestinal washes were assessed as before [12,14]. Briefly, a 20 cm long of enteral segment was cut, and the enteral interior was washed 3 times using 1 ml of cold PBS with 1% protease inhibitor (Sangon Biotech, Shanghai, China). The washing fluid was recovered, centrifuged at 10000 *g* for 5 min, and the supernatants were collected. Total enteral sIgA was assessed with a sandwich ELISA as previously described [35]. TsPPase-specific sIgA was measured using by a conventional indirect ELISA with 2.5 μg/ml of rTsPPase. The coloration with OPD and measurement of the absorbance at 492 nm were performed as previously reported [36]. All samples were in duplicate.

### Detection of cytokines by qPCR and ELISA

In order to detect the cellular immune response to rTsPPase immunization, the spleen, MLN and Peyer’s patches were isolated from immunized mice. The cells were prepared and cultured in RPMI-1640 medium which containing 5% FBS (fetal bovine serum, Gibco, New Zealand) the cell density were modulated to 5 × 10^6^ cells/ml and stimulated with 5 μg/ml rTsPPase for 3 days [10]. After cultivation, the levels of IFN-γ and IL-4 in RPMI-1640 medium were determined by sandwich ELISA and showed as pictograms per milliliter (pg/ml) [37]. Furthermore, the cDNA of these cells was extracted and used for qPCR to assay transcription levels of IFN-γ and IL-4 [38].

### Antibody-dependent cell-mediated cytotoxicity (ADCC) assay

The cytotoxicity of anti-rTsPPase serum against *T*. *spiralis* NBL was ascertained as previously described, the immune serum was from pooled serum of all animals immunized [39,40]. Briefly, 100 NBL were cultured with 2 × 10^5^ murine peritoneal exudate cells (PECs) in a 96-well plate with RPMI-1640 medium supplemented with anti-rTsPPase serum (1:10–1:500 dilutions) at 37°C for 72 h, infection serum was used as positive control, serum from the NC8 and MRS control mice as negative controls. After culture for 72 h, the larval viability was assessed according to their morphology and activity. The living NBL was active and movable, while the dead NBL was inactive and straight. Cytotoxicity was defined as the percentage of dead NBL to the total larvae observed in each test [41,42].

### Challenge infection with *T*. *spiralis* to evaluate immune protection

To assess the immune protection produced by oral rTsPPase vaccination, all vaccinated mice were orally infected with 300 *T*. *spiralis* ML at 2 weeks after the final vaccination. Ten mice from three groups were euthanized at 24 hpi, 6 and 35 dpi to collect the IIL, AW and ML, respectively [43,44]. The immune protection was ascertained according to mean number of intestinal 24 h IIL and 6 d AW and muscle larvae per gram (LPG) from recombinant NC8 group relative to those from the MRS medium control group [29,45]. The molting rate of 24 h IIL and length of intestinal worms were also measured to evaluate the inhibitory effect of recombinant NC8 immunization on larval molting and development. Furthermore, intestinal and masseter muscle samples of immunized were obtained to prepare 2-μm thick tissue sections, which were stained with hematoxylin and eosin (H&E) and periodic acid Schiff reagent (PAS; Baso, Zhuhai, China). The sections were examined under light microscopy, and the inflammatory cells (eosinophils, neutrophils and lymphocytes) and goblet cells per field (400×) were observed and numbered to evaluate the pathological change of intestine and muscles as previously described [46].

### Statistical analysis

The data in this study were statistically analyzed by SPSS 21.0. The data were shown as mean ± standard deviation (SD). Differences between various groups were analyzed by Chi-square test or one way ANOVA after being tested by Shapiro-Wilk’s test and Levene’s test to check the datum normality and homogeneity. Correlation analysis was used to analyze the relationship between ADCC cytotoxicity and anti-rTsPPase antibody dilution/culture times. *P* < 0.05 was used as statistically different level.

## Results

### The biological characteristics of recombinant *L*. *plantarum* NC8

**Fig 2:**
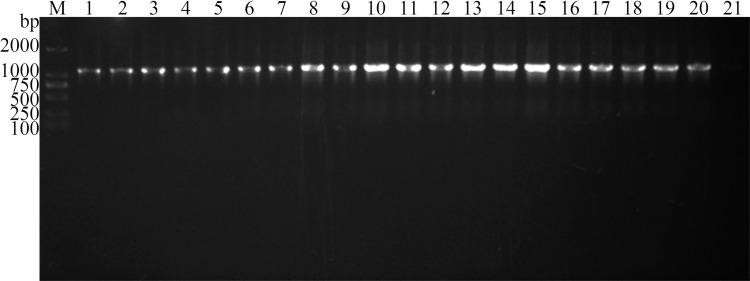
PCR identification of TsPPase in different generations of recombinant *L*. *plantarum* NC8. M: DNA marker; lane 1–20: PCR products of different generations of recombinant *L*. *plantarum* NC8; lane 21: products of normal *L*. *plantarum* NC8 (negative control).

**Fig 3:**
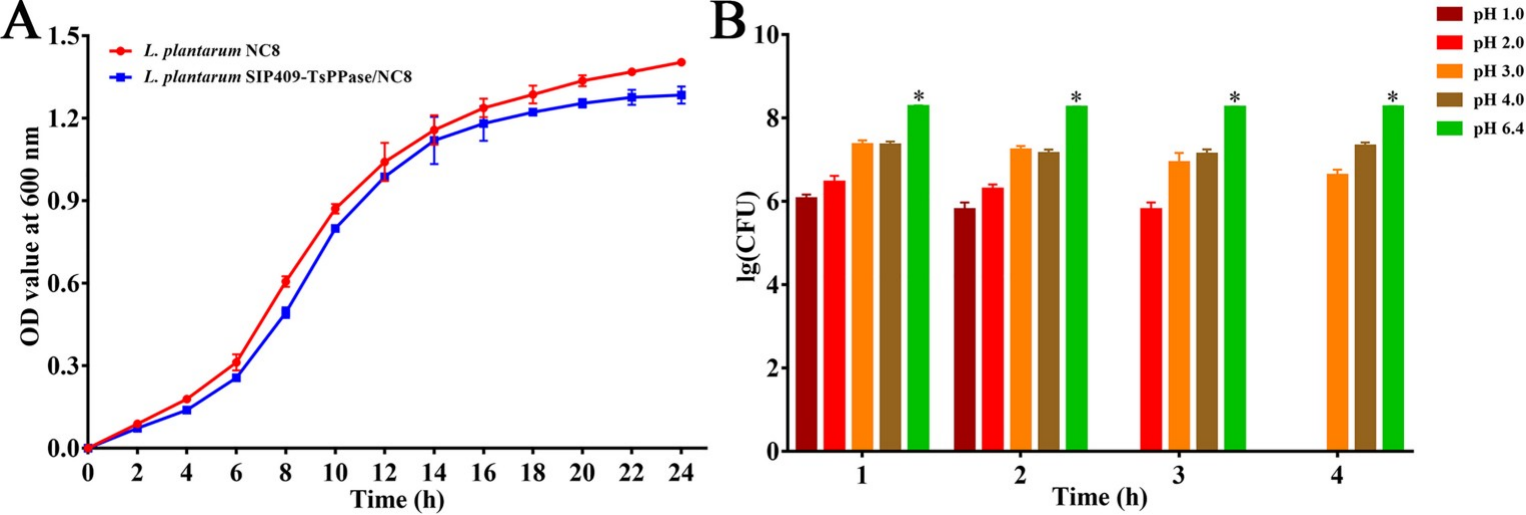
Biological characteristics of recombinant *L*. *plantarum* NC8. **A:** growth curve of recombinant *L*. *plantarum* NC8 and normal *L*. *plantarum* without pSIP409-pgsA′/TsPPase was used as the control. **B:** tolerance of recombinant NC8 in acid environment. Since the number of recombinant *L*. *plantarum* NC8 bacteria was too large, the data were compared by logarithm transform.

The results of the strain passage experiment showed that the pSIP409-pgsA′/TsPPase was detected in successive 20 generations of recombinant *L*. *plantarum* NC8, demonstrating that the pSIP409-pgsA′/TsPPase was stably inherited (Fig 2). The growth curve showed that the plasmid pSIP409-pgsA′/TsPPase had no inhibitory effect on proliferation of recombinant *L*. *plantarum* NC8, no significant difference was observed between the growth curve of recombinant and normal *L*. *plantarum* NC8 (Fig 3A) (t = 6.062, *P* = 0.116). The results of simulated gastric acid environment *in vitro* showed that recombinant *L*. *plantarum* NC8 survived for 2–3 hours in acidic environment (pH 1.0–2.0) and for a longer time in pH 3.0–4.0 environment. The number of recombinant bacteria in pH 1.0–4.0 was significantly lower than that in pH 6.4 (*F* = 243.031, *P* < 0.05) (Fig 3B).

### Expression of TsPPase in recombinant *L*. *plantarum* NC8

**Fig 4:**
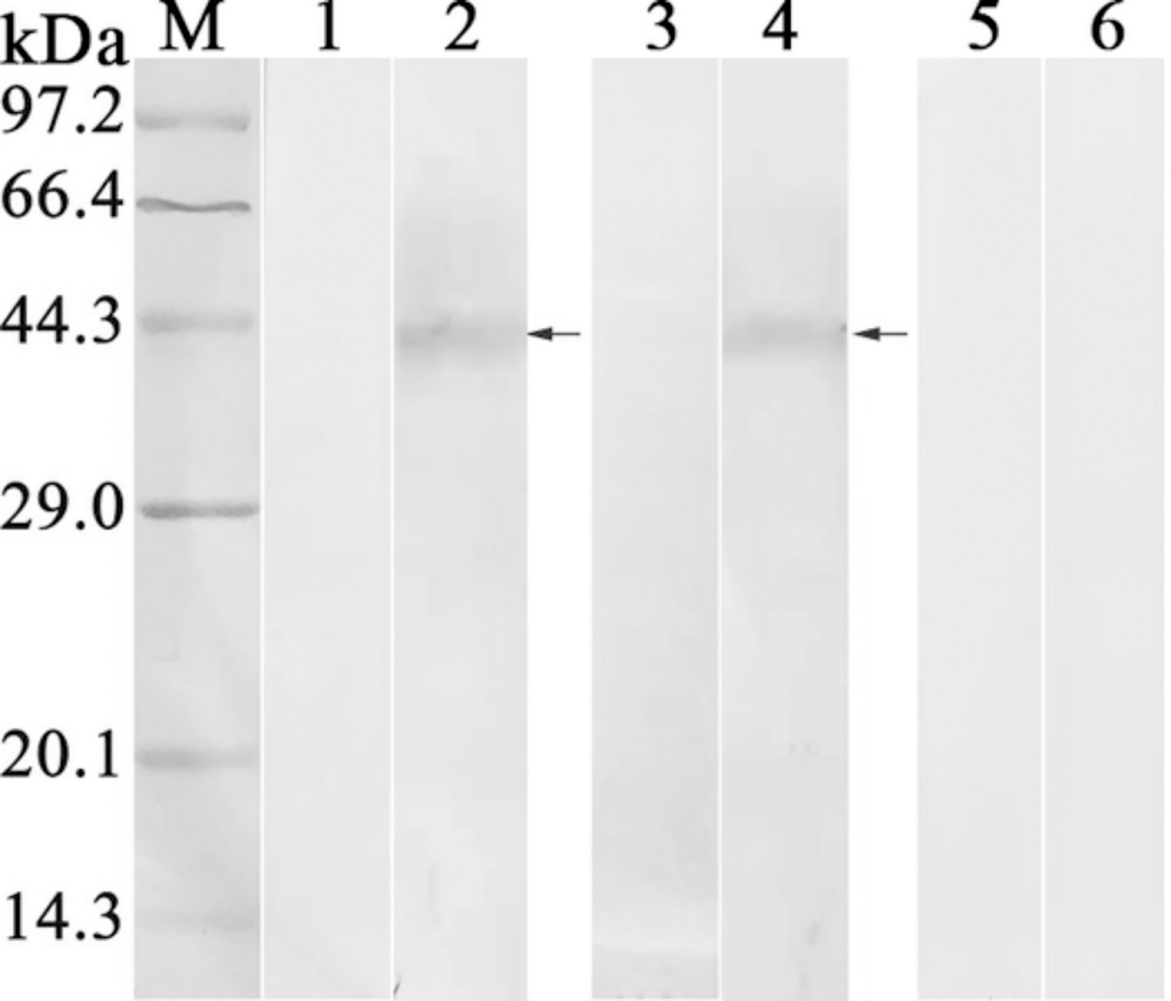
Western blot analysis of TsPPase expression. Soluble proteins of *L*. *plantarum* NC8 and recombinant NC8 were identified with infection serum (lane 1, 2), anti-rTsPPase serum (lane 3, 4) and normal serum (lane 5, 6), respectively. The recognized protein bands with about 44 kDa were indicated by arrows.

**Fig 5:**
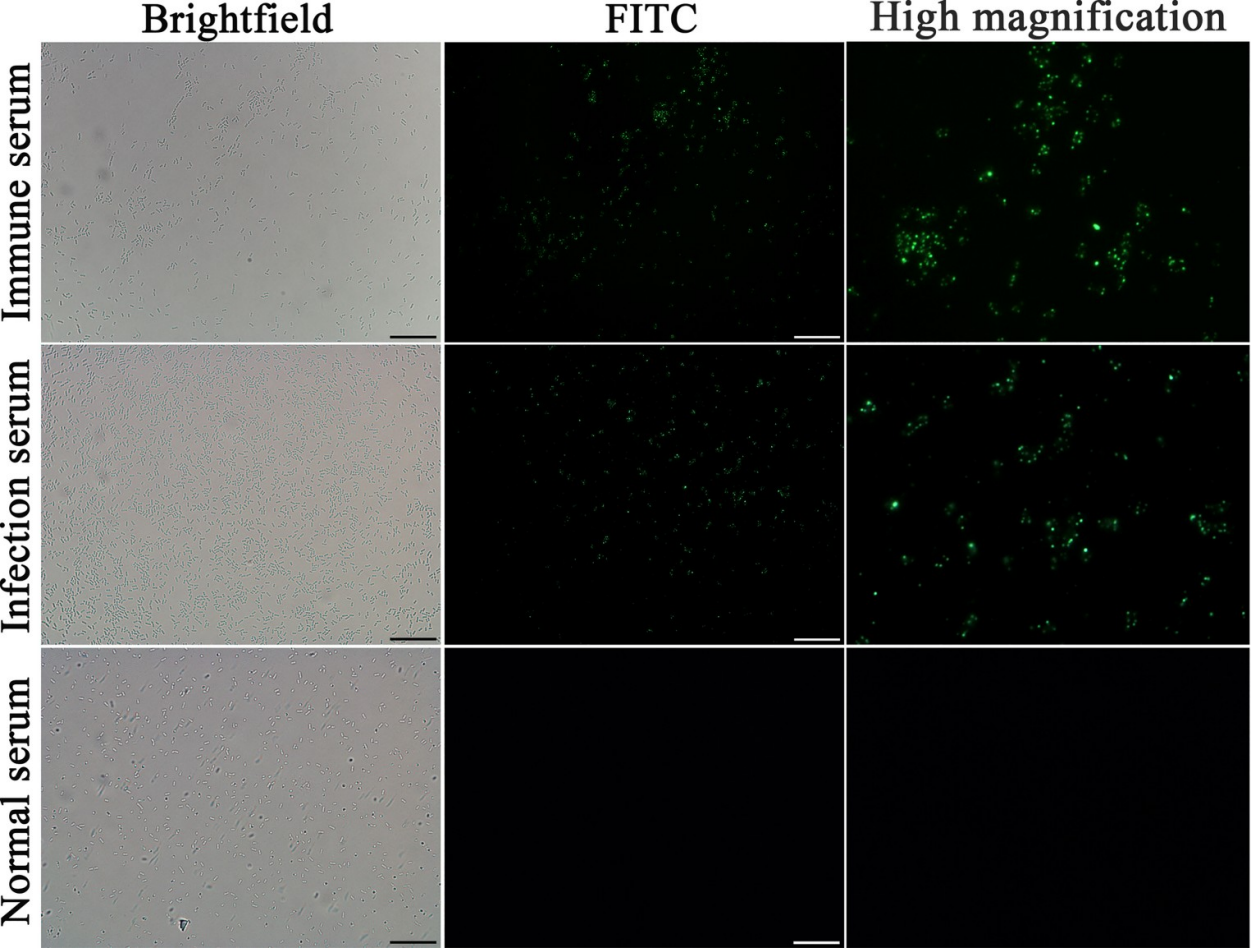
Immunolocalization of TsPPase on recombinant NC8 surface by IFA with anti-rTsPPase immune serum. Positive fluorescence staining was found on the surface of recombinant NC8 by using anti-rTsPPase serum. The bacteria recognized by infection serum as a positive control, and normal serum as the negative control. Scale bar: 5 μm.

The results of western blot revealed that an individual protein band of recombinant NC8 with about 44.0 kDa was recognized by anti-rTsPPase serum and infection serum, but no bands were recognized in the soluble protein of *L*. *plantarum* NC8 (Fig 4). Moreover, the IFA results showed that positive fluorescence staining was detected on the surface of recombinant NC8 by anti-rTsPPase immune serum and infection serum (Fig 5), indicating that the TsPPase protein was successfully expressed on the surface of recombinant NC8, but not expressed in normal *L*. *plantarum* NC8 without TsPPase gene.

### Expression of rTsPPase in intestinal epithelium of immunized mice by IFA

**Fig 6:**
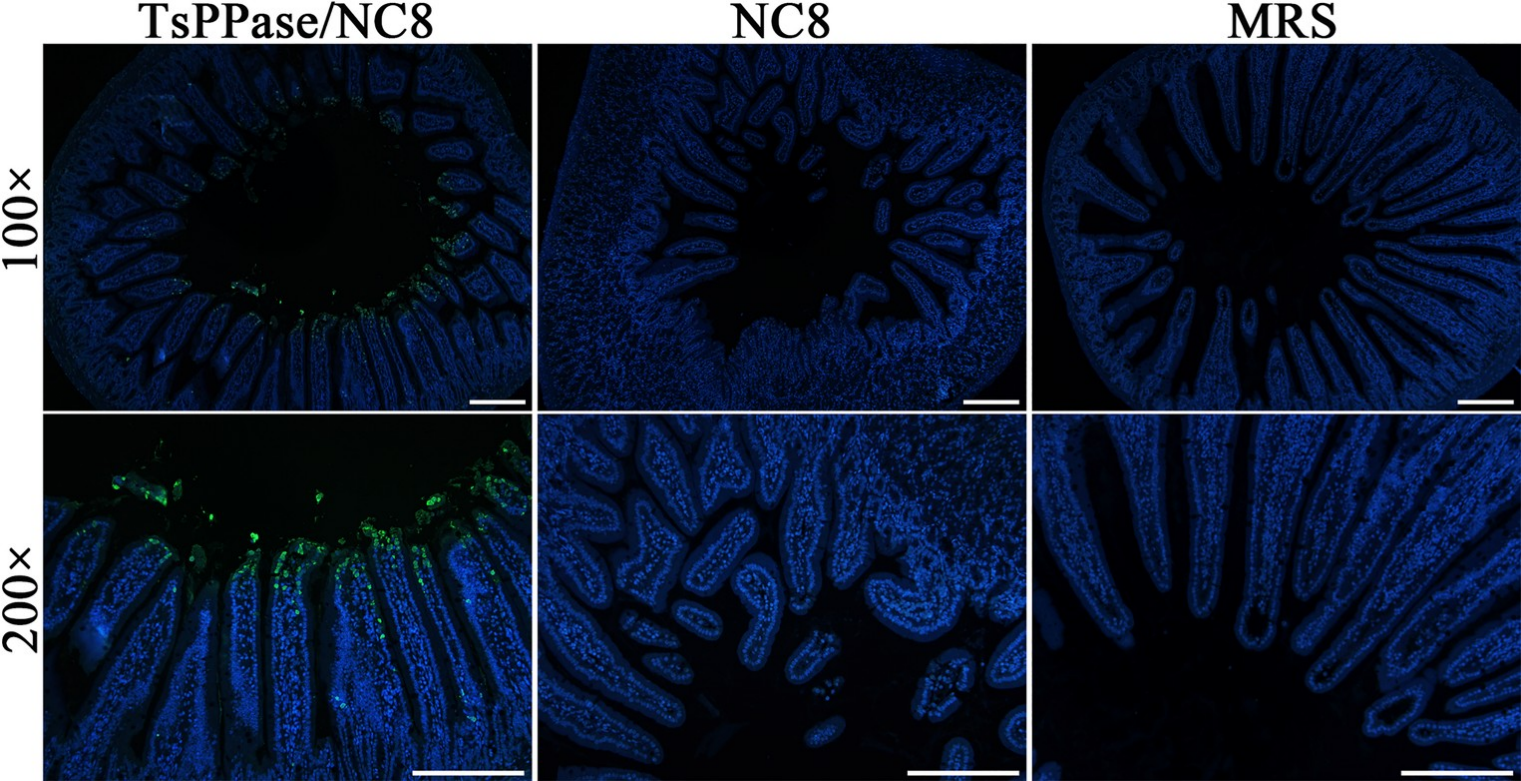
Expression of rTsPPase in intestinal epithelium of immunized mice by IFA. Positive green fluorescence staining was clearly observed in intestinal epithelium of mice immunized with recombinant NC8 group. There was no positive fluorescence staining in two control groups. The nuclei of intestinal cells were stained blue by 4′,6-diamidino-2-phenylindole (DAPI). Scale bar: 200 μm.

Intestine from immunized mice were embedded in paraffin, 2-μm thick intestinal sections were prepared using a microtome. IFA was performed with anti-rTsPPase serum. The result indicated that rTsPPase protein was detected in intestinal epithelium of mice immunized with recombinant NC8. But, no fluorescence staining was observed in intestinal sections of NC8 alone and MRS control mice (Fig 6).

### Serum anti-rTsPPase antibody response to rTsPPase immunization

**Fig 7:**
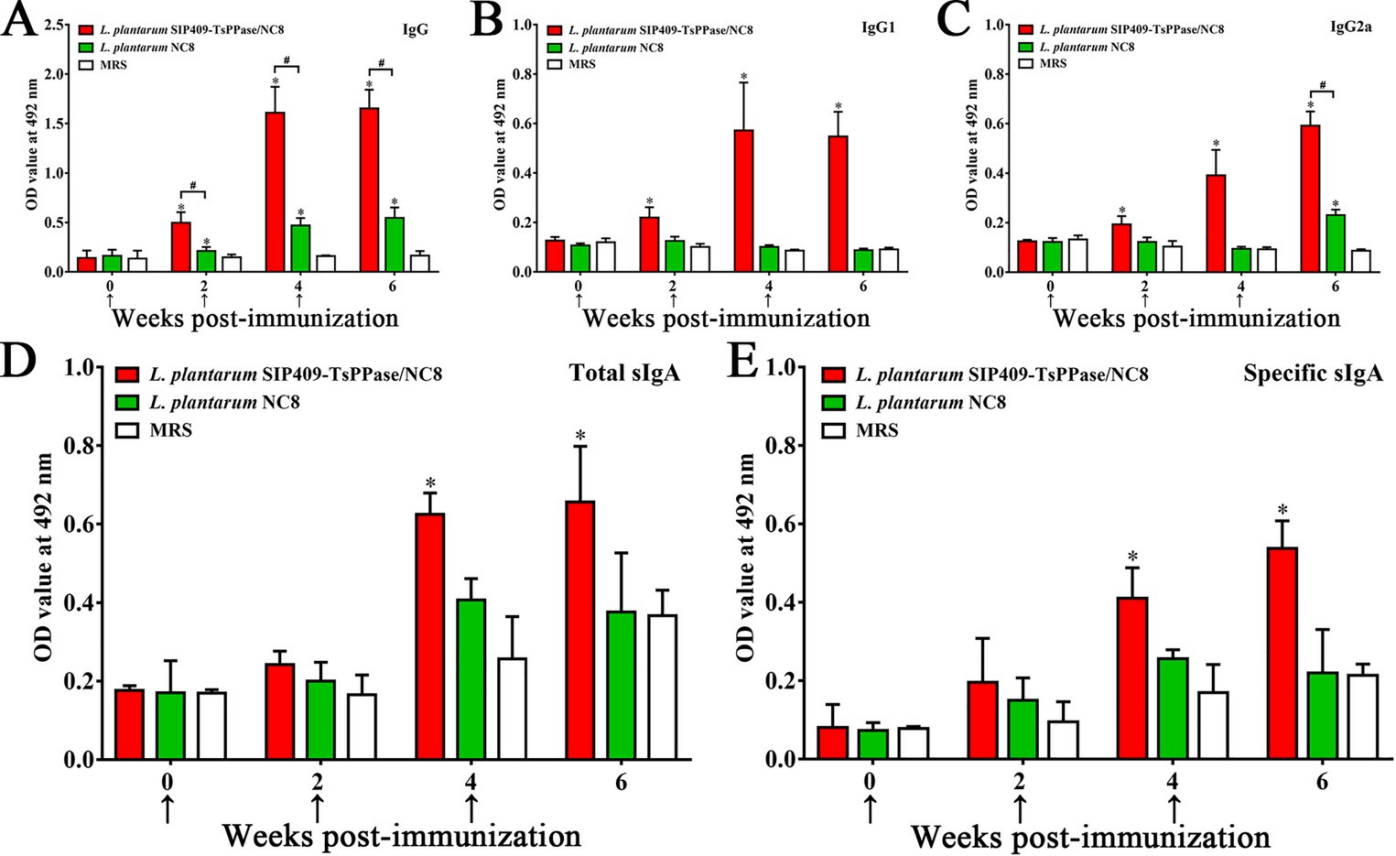
ELISA detection of anti-rTsPPase IgG and sIgA in mice orally immunized with recombinant NC8. **A:** Serum anti-rTsPPase IgG in vaccinated mine at diverse time intervals after immunization was assessed (n = 20). The IgG1 (**B**) and IgG2a (**C**) subclass response of immunized mice at diverse time intervals after immunization (n = 20). Total sIgA (**D**) and specific sIgA (**E**) levels of in recombinant NC8 group were obviously higher than NC8 and MRS groups (n = 5). The vaccination times are showed with arrowheads (**↑**). *P < 0.05 compared to the MRS group, ^#^*P* < 0.05 compared to the NC8 control group.

The ELISA results showed that the levels of anti-rTsPPase antibodies in serum of recombinant NC8 group were significantly increased following two immunization continuously. The total IgG levels of recombinant NC8 group were significantly higher than that of two control groups at 2, 4 and 6 weeks after vaccination (*F*_2W_ = 147.9, *F*_4W_ = 469.0, *F*_6W_ = 734.4, *P* < 0.0001); both IgG1 and IgG2a levels were also obviously higher than two control groups at 4 and 6 weeks following vaccination (*P* < 0.05) (Fig 7A–7C), but, there were no statistical difference between IgG1 and IgG2a levels (*t* = 1.734, *P* = 0.0911) indicating that recombinant NC8 immunization triggered a mixed Th1/Th2 immune response. No mice orally immunized with MRS exhibited any anti-rTsPPase IgG responses.

The results of sIgA assay revealed that total sIgA level in recombinant NC8 immunized mice was evidently higher than NC8 and MRS control groups (*F*_4W_ = 19.78, *F*_6W_ = 8.671, *P* < 0.05) (Fig 7D). Moreover, the specific sIgA level in recombinant NC8 group was also obviously higher than two control groups (*F*_4W_ = 19.0, *F*_6W_ = 28.49, *P* < 0.05) (Fig 7E). No specific enteral sIgA was detected in mice immunized with empty NC8 and MRS, suggesting that recombinant NC8 immunization elicited specific enteral local mucosal sIgA response.

### Expression levels of cytokines from immunized mice

**Fig 8:**
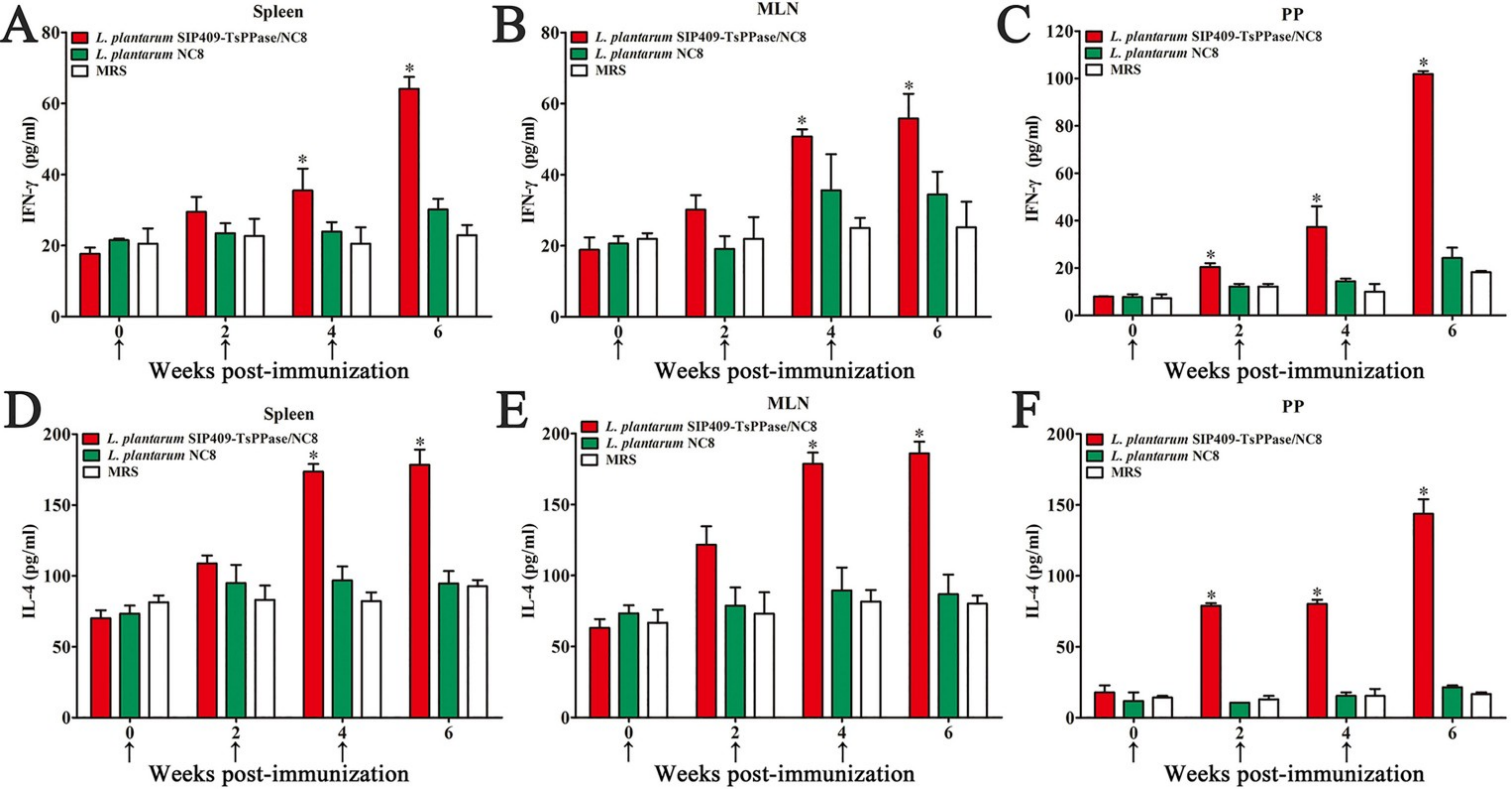
Levels of IFN-γ and IL-4 secreted by spleen, MLN and Peyer’s patch cells by sandwich ELISA. The data are shown as the mean ± SD of five animals/group. The vaccination time was signed with an arrow (↑). The detection limit of IFN-γ was 15–2000 pg/ml, and that of IL-4 was 2–250 pg/ml. *P < 0.05 compared to the empty NC8 and MRS medium control group.

**Fig 9:**
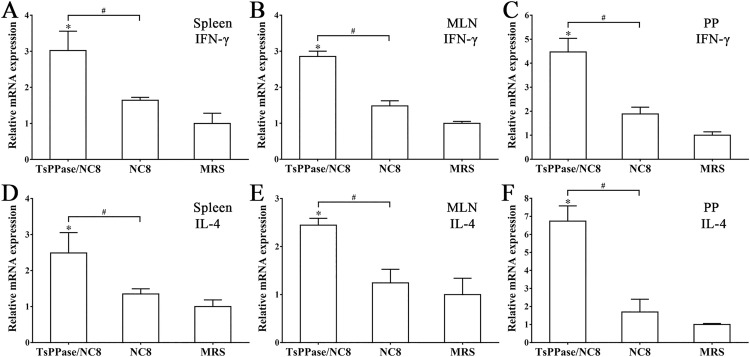
Transcription levels of IFN-γ and IL-4 secreted by spleen, MLN and Peyer’s patch cells. The cDNA of spleen, MLN and Peyer’s patch cells was extracted and used for qPCR to assay transcription levels of IFN-γ and IL-4 at two weeks after the third immunization. *P < 0.05 compared to MRS, ^#^*P* < 0.05 compared to NC8 control group.

After the first immunization, the levels of IFN-γ and IL-4 produced by Peyer’s patch cells were obviously higher than the NC8 and MRS control groups (*F*_IFN-γ_ = 13.24, *P* < 0.05; *F*_IL-4_ = 13.359, *P* < 0.05). The levels of IFN-γ and IL-4 secreted by spleen and MLN cells were evidently higher than two control groups following the second immunization (*P* < 0.05) (Fig 8). The qPCR results also showed that transcription levels of IFN-γ and IL-4 in recombinant NC8 immunized group were significantly higher than that of NC8 and MRS control groups (*P* < 0.05) (Fig 9). The results indicating that oral immunization with recombinant NC8 triggered both the systemic (spleen) and enteral mucosal local (MLN and Peyer’s patches) cellular immune response.

### The cytotoxicity of ADCC assay

**Fig 10:**
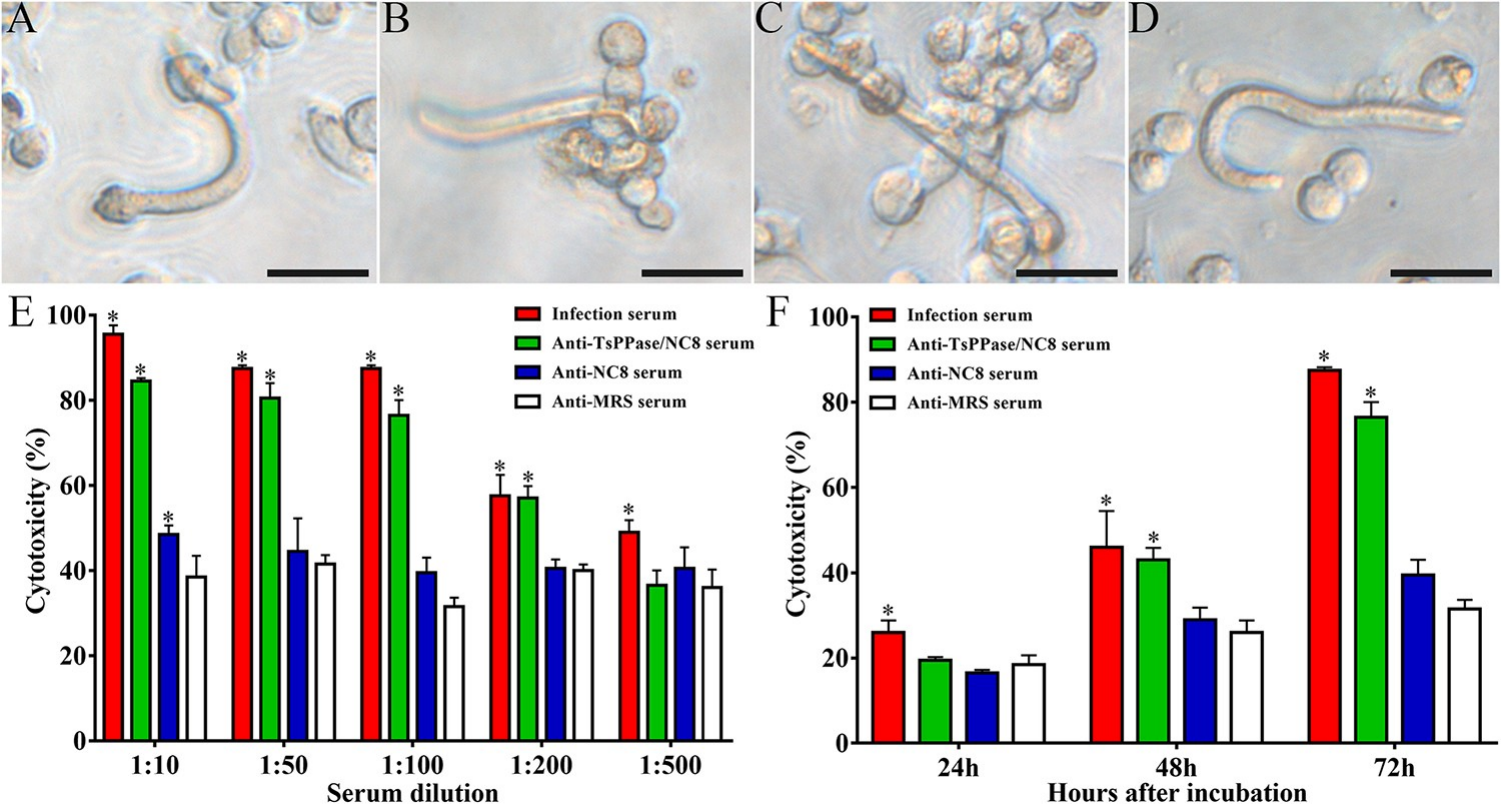
Killing effect of anti-rTsPPase antibody mediated ADCC on NBL. The number of PECs adhering to NBL was increased at 24 (**A**), 48 (**B**) and 72 h (**C**) in anti-rTsPPase antibody mediated ADCC test. There was no killing effect in ADCC without serum (**D**). **E:** The cytotoxicity of ADCC was dose-dependent of anti-rTsPPase antibodies. **F:** Cytotoxicity was increased with prolonged culture time. *P < 0.05 compared to serum from the NC8 and MRS control mice. Scale bars: 50 μm.

The *in vitro* ADCC assay was performed, and the result showed that anti-rTsPPase serum had the ability to promote PECs adhesion to NBL (Fig 10A–10C). There was no PECs adhesion to NBL in the absence of anti-rTsPPase serum (Fig 10D). When the dilution of anti-rTsPPase serum was 1:10, 1:50, 1:100 and 1:200, the larval mortality was 84.5%, 80.5%, 76.5% and 57% respectively, significantly higher than that of serum from the NC8 and MRS control mice (*F*_1:10_ = 178.255, *F*_1:50_ = 58.462, *F*_1:100_ = 200.422, *F*_1:200_ = 10.304, *P* < 0.05) (Fig 10E), indicating that the cytotoxicity decreased with the increasing serum dilution (*F* = 33.056, *P* < 0.05, r = 0.985). Furthermore, the cytotoxocity of anti-rTsPPase serum (1:100 dilution) at 48 and 72 h after culture was obviously stronger than that of serum from the NC8 and MRS control mice (*F* = 200.422, *P* < 0.05); larval mortality at 24, 48 and 72 h after culture was 19.5%, 43.0% and 76.5%, respectively, the cytotoxicity had an increasing trend following the extension of culture times (*F* = 137.839, *P* < 0.05, r = 0.993) (Fig 10F).

### Immune protection of recombinant NC8 immunization

**Fig 11:**
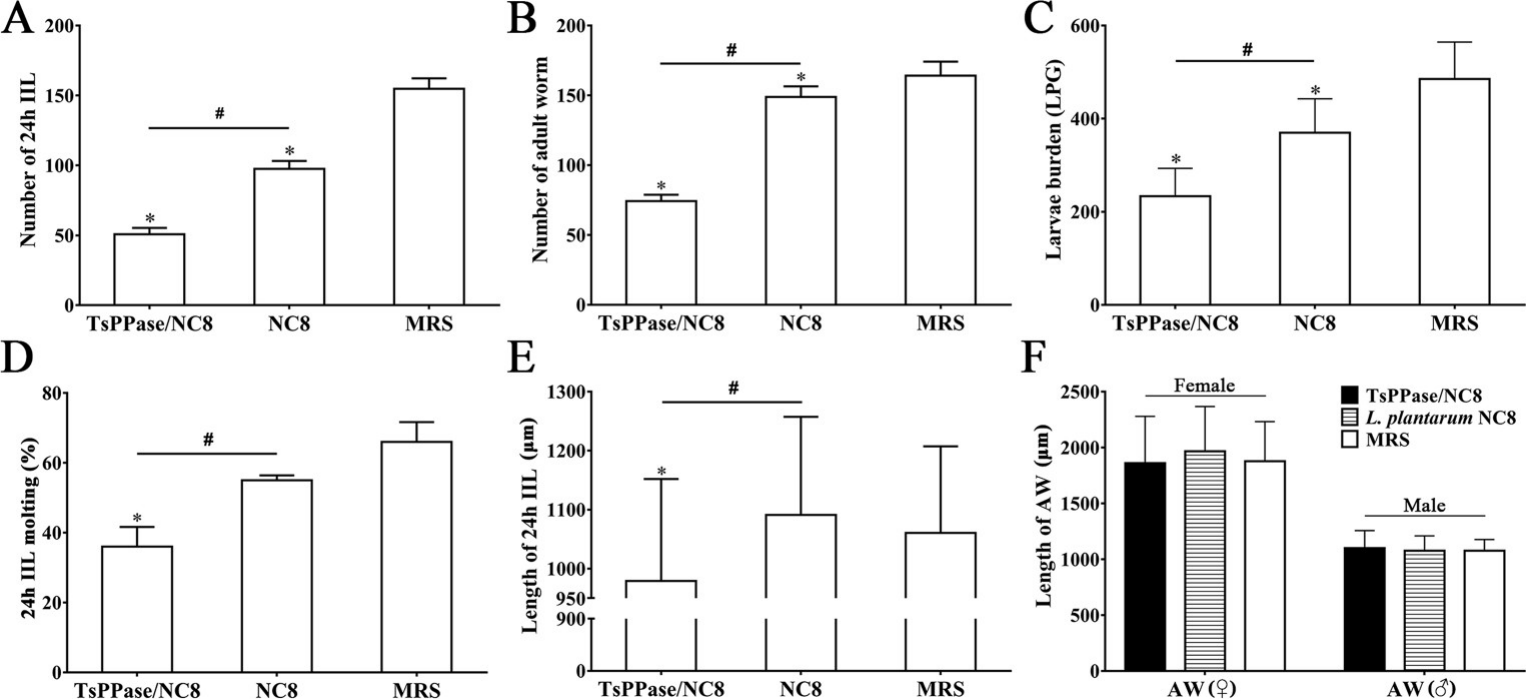
Inhibition of recombinant NC8 immunization on intestinal *T*. *spiralis* larval molting and development. Comparison of the number of 24 h IIL (**A**), 6 d AW (**B**) and 35 d ML (**C**) collected from the 3 groups of mice infected with 300 *T*. *spiralis* larvae. **D:** Larval molting of 24 h IIL. **E:** Length of 24 h IIL. **F:** Length of 6d male and female AW. *P < 0.05 compared to the MRS medium group, ^#^*P* < 0.05 compared to the normal NC8 control group.

**Fig 12:**
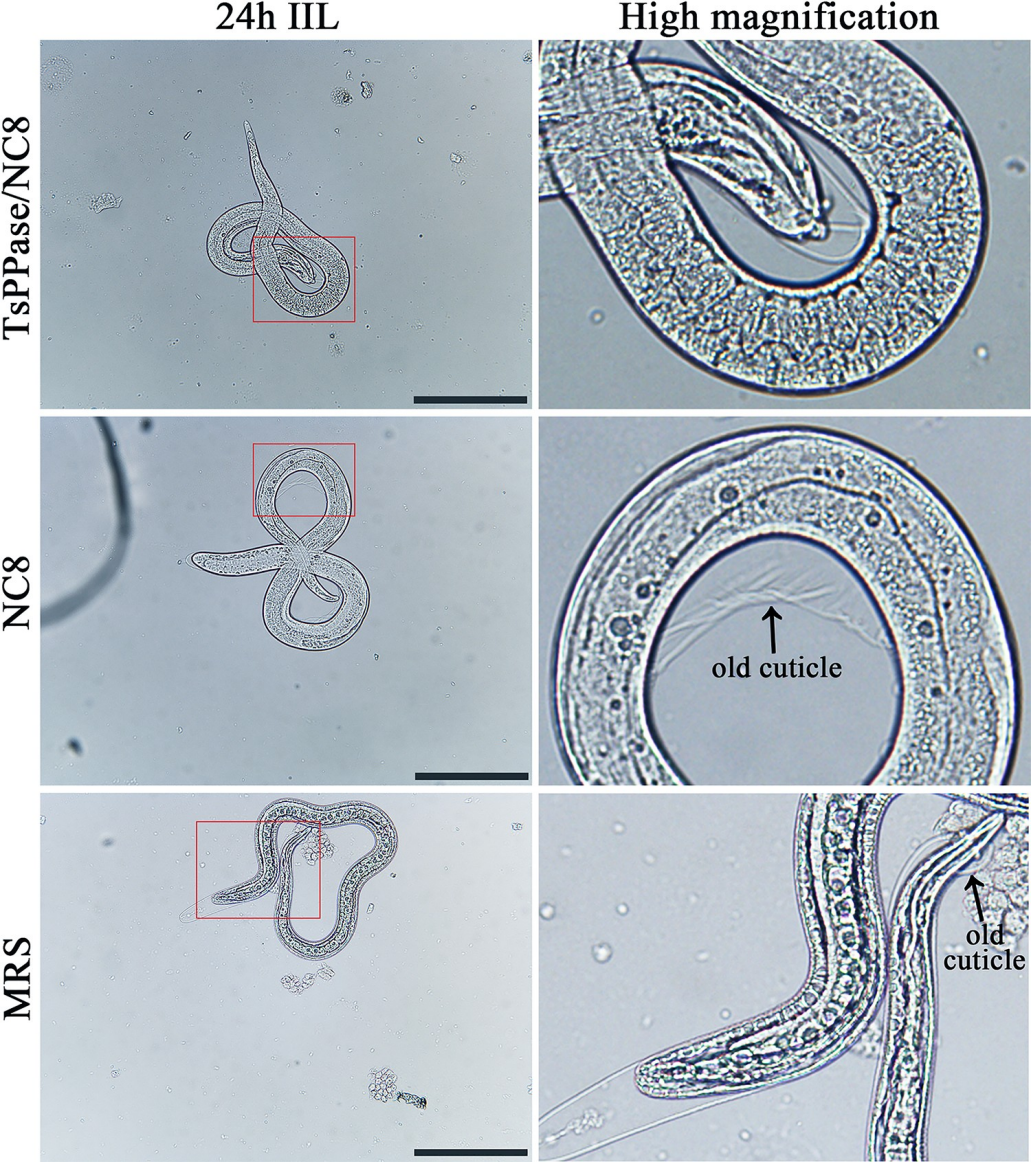
Intestinal larval molting was suppressed by recombinant NC8 immunization. The larval molting of 24 h IIL from recombinant NC8-immunized mice was obviously inhibited, no obvious separation between the old and new cuticle was observed except the larval tail end. However the isolation of old and new cuticles of 24 h IIL from NC8 and MRS control groups was clearly observed. The area in the red box was enlarged for observation (Scale bar: 200 μm).

Compared to the MRS medium group, the recombinant NC8-immunized mice exhibited a 67.18, 54.78 and 51.91% reduction of IIL, AW and ML, respectively (*F*_IIL_ = 755.480, *F*_AW_ = 386.043, *F*_ML_ = 17.717, *P* < 0.05), whereas empty NC8 control group exhibited only a 36.95, 9.32 and 23.84% reduction of IIL, AW and ML, respectively (*P* < 0.05) (Fig 11A–11C), suggesting that although oral immunization with empty NC8 elicited a partial protection against *T*. *spiralis* infection, the protective effect was apparently lower than that of recombinant NC8 immunization. The larval molting rates of 24 h IIL were also assessed, the larval molting of 24 h IIL from recombinant NC8 group was obviously inhibited (Figs 11D and 12). The larval molting rate (36%) of recombinant NC8 group was significantly lower than the MRS control group (66%), larval molting was inhibited by 45.45% (χ^2^ = 18.007, *P* < 0.05). Intestinal larval development was also impeded, the length of 24 h IIL from recombinant NC8 group was significantly shorter than two control groups (*F* = 12.707, *P* < 0.05) (Fig 11E). However, there was no statistical difference among the AW length in three groups (*P* > 0.05) (Fig 11F), suggesting that recombinant NC8 immunization suppressed intestinal *T*. *spiralis* larval molting and development, but there were no effects on adult growth after larval ecdysis. The vaccine efficacy was estimated by 100 × (1-IRR) where IRR is the calculated ratio of collected 6 d AW burden in the recombinant NC8 group to the corresponding 6 d AW burden in the MRS group. The vaccine efficacy was 54.78%.

### Histopathological changes of intestine and skeletal muscles in immunized mice

**Fig 13:**
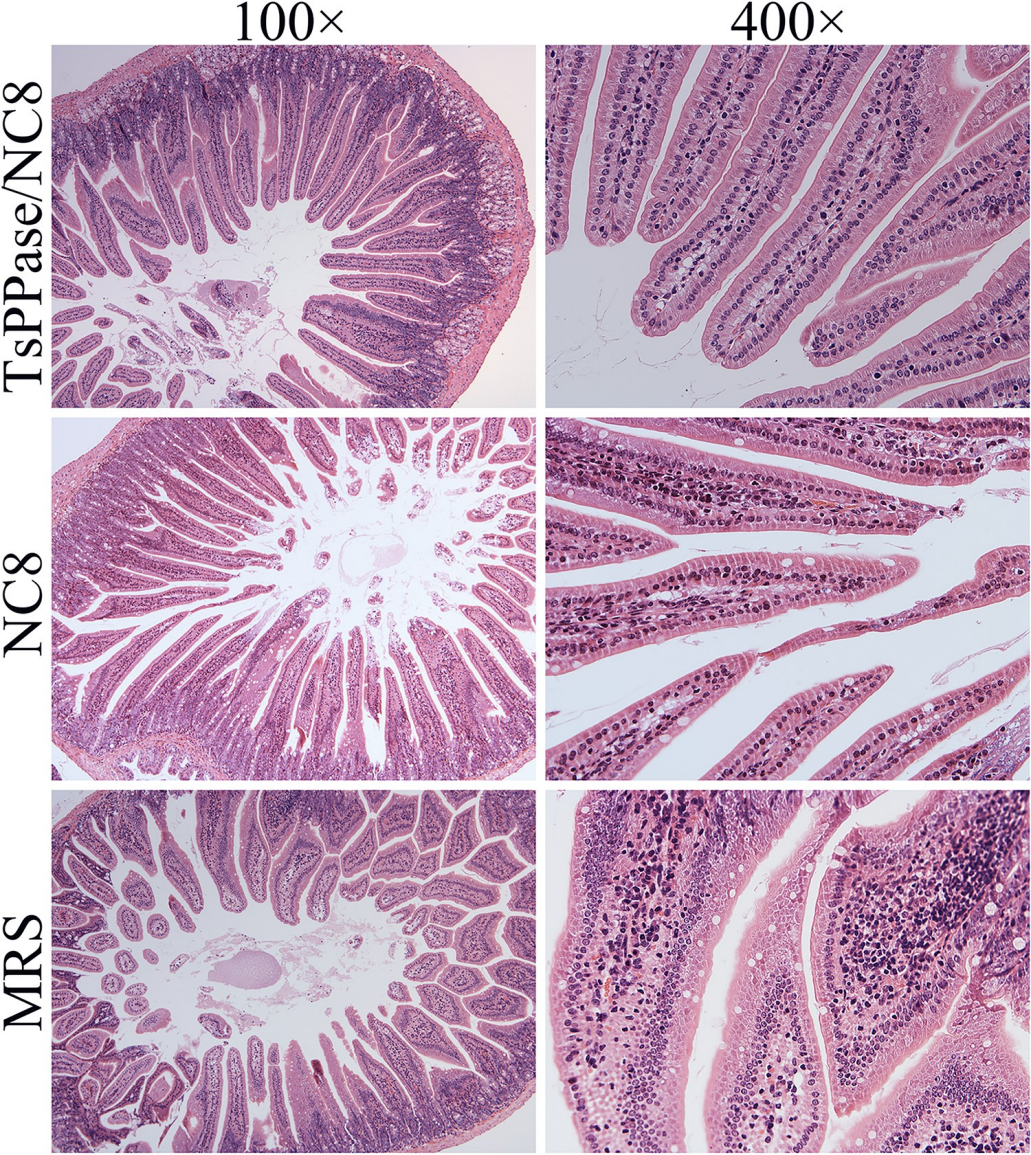
Intestinal histopathological changes in immunized mice at 6 days after *T*. *spiralis* infection. Intestinal sections were stained using haematoxylin and eosin (HE) and examined under light microscopy. Intestinal pathological changes from recombinant NC8 immunized mice and NC8 control mice were significantly alleviated. Serious intestinal inflammation and shortened intestinal villi were observed in intestinal section of MRS medium control mice.

**Fig 14:**
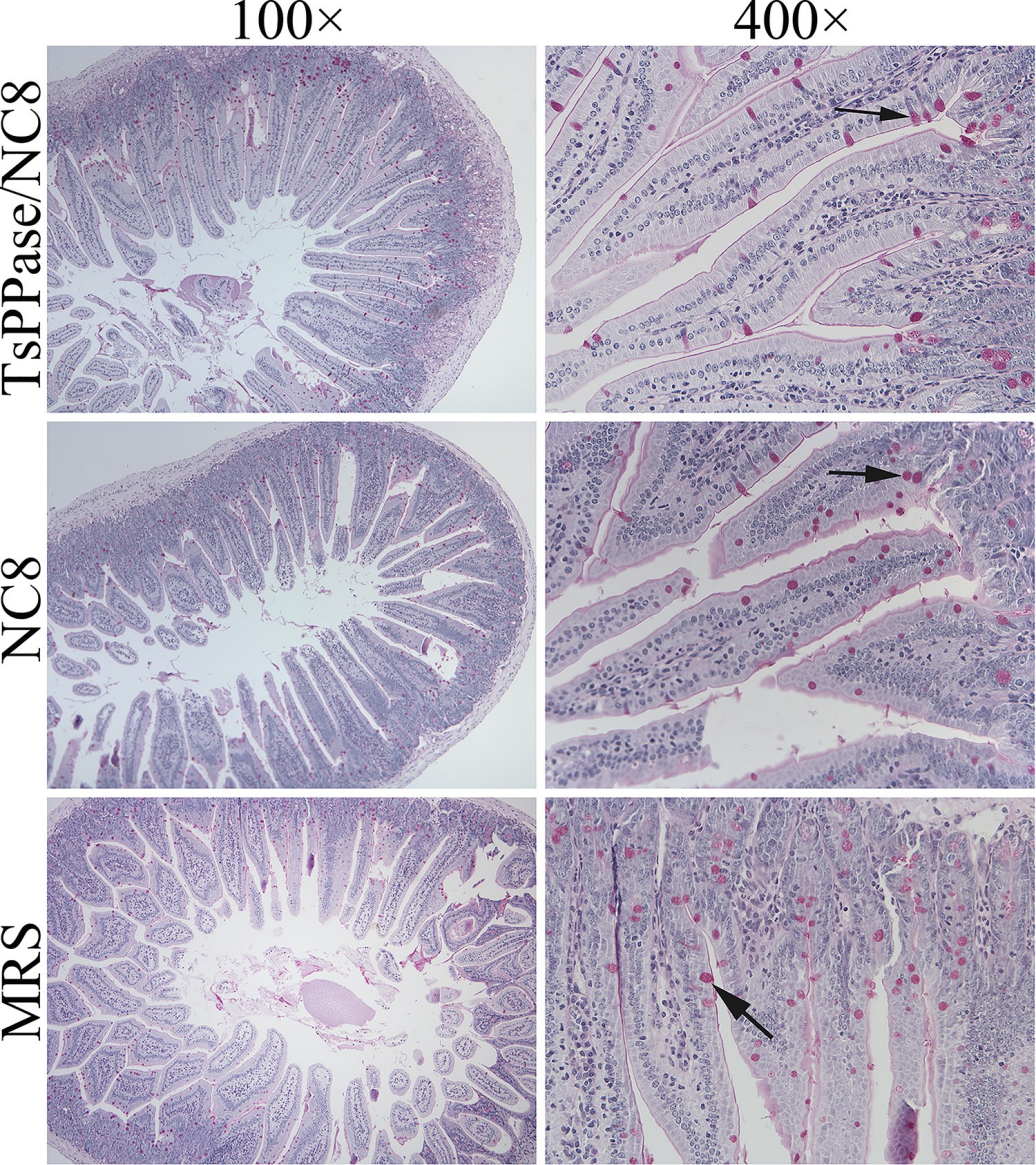
PAS staining of intestinal sections from immunized mice at 6 days after *T*. *spiralis* infection. The number of goblet cells in recombinant NC8-immunized mice was evidently reduced compared to the MRS medium control mice, in which small intestine was seriously intruded and infected with *T*. *spiralis*. Goblet cells were indicated by solid arrows.

**Fig 15:**
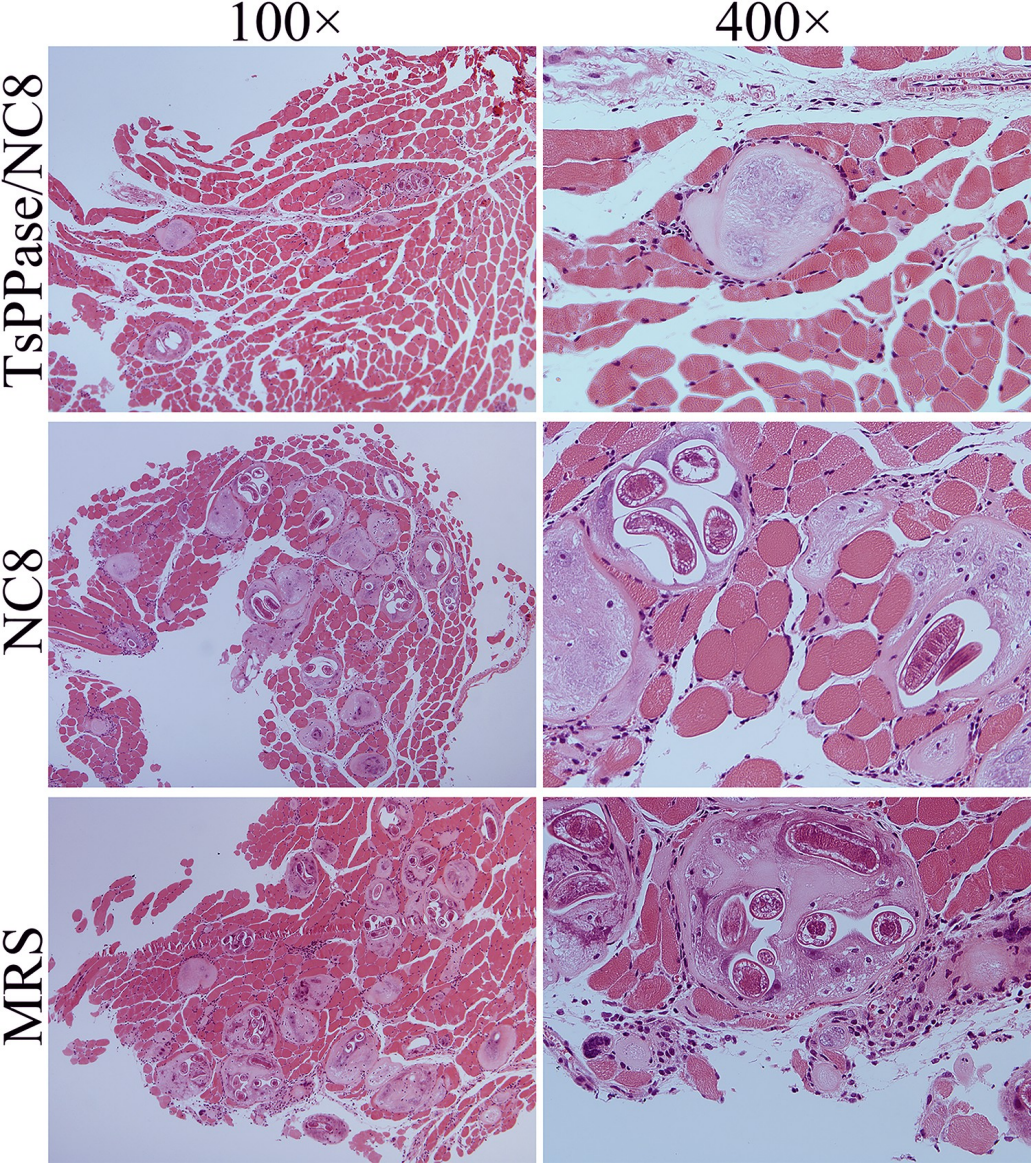
Muscle histopathological changes in immunized mice at 35 days after *T*. *spiralis* infection. Mild inflammatory reaction and less encapsulated muscle larvae were observed in muscle section of recombinant NC8-immunized mice.

**Fig 16:**
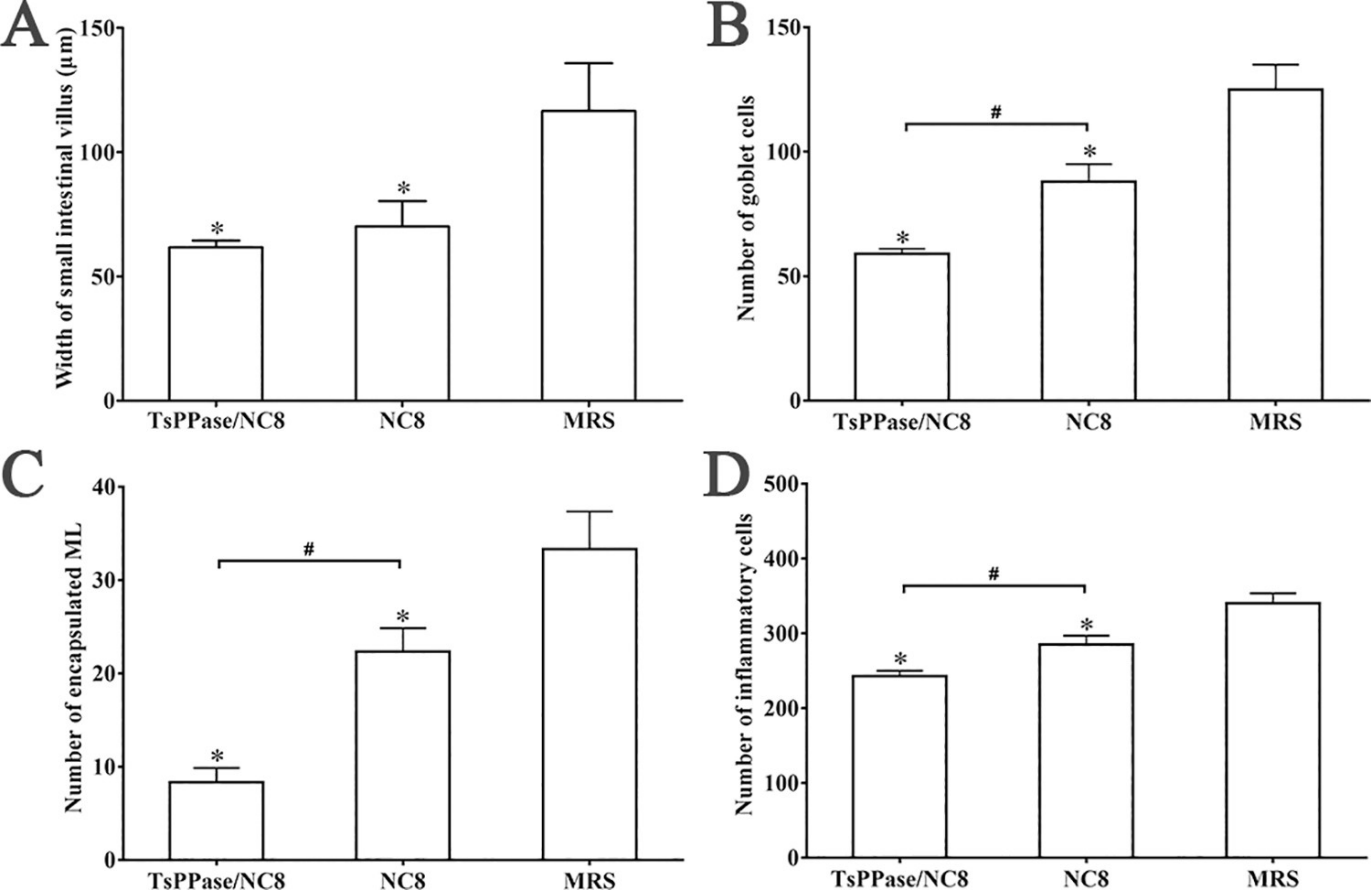
Enteral and muscle pathological changes in immunized mice at 6 and 35 dpi. **A:** Intestinal villus width at 6 dpi. **B:** Number of intestinal goblet cells at 6 dpi. **C:** Number of encapsulated *T*. *spiralis* muscle larvae (ML) at 35 dpi. **D:** Number of inflammatory cells around encapsulated larvae on muscle sections at 35 dpi. *P < 0.05 compared to MRS, ^#^*P* < 0.05 compared to NC8 control group.

Histopathological changes of intestine and skeletal muscles from immunized mice were examined at 6 and 35 dpi, respectively. As shown in Figs 13 and 14, after *T*. *spiralis* challenge infection, mild intestinal inflammation and normal intestinal villi were observed in intestinal section of recombinant NC8-immunized mice, and the number of goblet cells reduced significantly (Fig 14), indicating that recombinant NC8-immunization obviously impeded larval invasion and alleviated intestinal inflammation (*F* = 64.37, *P* < 0.05). The results of HE staining of muscle tissue showed mild inflammatory reaction and less encapsulated ML were observed in muscle section of recombinant NC8-immunized mice (Figs 15 and 16).

## Discussion

Inorganic pyrophosphatase (PPase) is a kind of enzymes with catalytic activity, which mainly plays a role in the synthesis of biological macromolecules in life. PPase can catalyze the hydrolysis of inorganic pyrophosphate (PPi) into two molecules of phosphate (Pi) to control the concentration of pyrophosphate in cells and regulate the intracellular water-salt balance; the degradation of ATP was also catalyzed by PPase to provide energy for life activities [47]. Studies on parasites showed that PPase participated in the development process and played a crucial role in parasite survival, growth and larval molting [48,49]. In our previous study, TsPPase was identified and its role in *T*. *spiralis* life cycle was ascertained. TsPPase participated in *T*. *spiralis* larval molting, the larval molting and development of *T*. *spiralis* were obviously suppressed when TsPPase was inhibited by inhibitor NaF and specific RNA interference (RNAi) [22]. These results indicated that TsPPase had the potential to be a promising target for anti-*Trichinella* vaccine, thus the protective immunity of rTsPPase against *T*. *spiralis* infection was investigated in the present study.

Lactic acid bacteria (LAB) have been recognized as a probiotic and harmless to humans and other animals. LAB was used as a good carrier for the construction of immune protective vaccines because of its probiotic effect [50,51]. A recombinant *L*. *plantarum* containing gp85 gene of J Subgroup Avian Leukosis Virus (ALV-J) was constructed, the results showed that oral immunization with recombinant *L*. *plantarum*/gp85 provided an effective mean for eliciting protective immune response against early ALV-J infection [52]. The *L*. *plantarum* was also used to construct a recombinant vaccine and orally immunized mice to evaluate the efficiency of preventing pathogenic avian influenza, the mice were protected against infection challenge with the H9N2 and H1N1 viruses [53]. Immunization with recombinant *L*. *plantarum* in chicken also improved humoral and cellular immunity and enhanced resistance to *Eimeria tenella* [54]. The results showed that recombinant vaccines constructed by LAB had a good immune protection. Therefore, the *L*. *plantarum* NC8 was selected to construct the recombinant TsPPase vaccine in this study. Moreover, previous studies revealed that the pgsA’ surface display module was effective for anchored expression of external proteins and it could promote the protein expression, thus the pSIP409-pgsA′ plasmid was used to construct the recombinant TsPPase plasmid to ensure the expression of rTsPPase [55].

In this study, biological characteristics of recombinant TsPPase/NC8 were firstly assessed, the results showed that the pSIP409-pgsA′/TsPPase was stably inherited and the recombinant NC8 could survive under acidic conditions. The results of western blot and IFA revealed that rTsPPase protein was successfully expressed on the surface of recombinant NC8. After immunization with recombinant NC8 vaccine, the levels of specific antibodies in serum (IgG, IgG1 and IgG2a) and intestinal fluid (sIgA) were obviously increased. The results of IgG subclass and cytokines in immunized mice demonstrated that oral administration of recombinant NC8 elicited a Th1/Th2 mixed immune response to TsPPase. T cell immune responses play a crucial role in prevention of pathogen infection, the Th1 cells secret the IFN-γ, activate macrophages and enhance their ability to kill the phagocytized pathogens, it can also promote the production of IgG [56]. The major roles of Th2 cells are to assist the activation of B lymphocyte. The cytokines such as IL-4 secreted by Th2 cells can promote the proliferation, differentiation and antibody production of B lymphocyte; it mainly participates in the immune responses and tissue repair process against *T*. *spiralis* [57]. Our results of ADCC assay showed that TsPPase-specific antibodies promoted macrophage adherence and killing to the NBL, and the cytotoxicity was dose-dependent of anti-TsPPase antibodies [58].

The results of challenged infection showed that *T*. *spiralis* larval molting and development were prominently suppressed in intestine of recombinant NC8-immunized mice, the length of 24 h IIL was obviously shorted than the normal NC8 and MRS control groups. The immunized mice exhibited a 67.18, 54.78 and 51.91% reduction of 24 h IIL, 6 d AW and 35 d ML, whereas the NC8 control mice exhibited only a 36.95, 9.32 and 23.84% reduction of 24 h IIL, 6 d AW and 35 d ML compared with MRS group (*P* < 0.05). Furthermore, recombinant NC8-immunization obviously alleviated intestinal inflammation, and intestinal pathological changes were also mitigated in normal NC8 control mice, indicating oral administration of normal *L*. *plantarum* NC8 provided a partial protection from *T*. *spiralis* invasion. However, intense intestinal inflammation, shortened intestinal villi and more encapsulated ML were observed in the MRS medium control mice, suggesting that the mice inoculated with only MRS medium were seriously infected by *T*. *spiralis*. The number of inflammatory cells reflects the intensity of intestinal inflammatory response, the more inflammatory cells, the more serious the *T*. *spiralis* infection [59]. Goblet cells are mucus secreting cells which mainly distributed among the columnar epithelial cells, they promote worm discharge from guts by secreting mucus, the number of goblet cells is positively correlated with the severity of *T*. *spiralis* infection in mice, the notable proliferation of goblet cells indicates that mice are seriously infected by *T*. *spiralis* [60,61]. sIgA plays a key role in intestinal mucosal immunity, sIgA is structurally equipped to resist chemical degradation in the harsh environment of mucosal surfaces and enzymes of host or microbial origin. Most infectious pathogens enter the host via mucosal surfaces, and the sIgA is the first line of protection at these entry ports [62]. In this study, the recombinant NC8/TsPPase vaccination elicited high level of TsPPase-specific mucosal sIgA production, which could accelerate adult worm ejectment from the guts and suppressed the reproductive ability of *T*. *spiralis* female adult worms, as a result, reduced larval burdens in muscles [63,64]. Our results showed that recombinant NC8-immunization promoted discharge of intestinal larvae and adult worms, and improved intestinal mucosal inflammation [25].

Previous studies showed that the mice immunized with a recombinant single *T*. *spiralis* protein could not completely eliminate *T*. *spiralis* infection. Subcutaneous vaccination of mice with rTsSerp or rTsASP1 exhibited a 52.5% or 50.55% muscle larval reduction after *T*. *spiralis* larval challenge [9,10]. When attenuated *Salmonella* was used as the carrier of TsPmy and TsDNase II DNA vaccine, the muscle larval reduction was 46.6 and 59.26%, respectively [13,15]. In the present study, although oral vaccination with recombinant NC8/TsPPase induced specific humoral and mucosal immune responses, only a 51.91% of larval reduction in muscles tissues was observed following challenge, *T*. *spiralis* larvae were not entirely eradicated in vaccinated animals. The results demonstrated that vaccination with an individual *Trichinella* protein molecule only produced a partial immune protection against challenge infection. *Trichinella spiralis* is a multicellular zoonotic parasitic nematode with a complex life cycle, each life cycle stage has its stage-specific antigens [65,66]. Therefore, to eliminate *Trichinella* larvae in food animal, the multivalent anti-*Trichinella* vaccines composed of various protective antigenic epitopes need to be developed [67]. Moreover, other immunization strategies including heterologous prime-boost vaccination and different adjuvants are necessary to be explored [68].

In conclusion, a recombinant *L*. *plantarum* NC8/TsPPase was prepared in this study. The rTsPPase protein was expressed on the surface of recombinant NC8. Oral vaccination of mice with recombinant NC8/TsPPase DNA vaccine triggered a systemic concurrent Th1/Th2 immunity as well as enteral local mucosal responses, and a significant immune protection against *T*. *spiralis* infection. The aim of the study was to investigate a safe and effective mean to reduce *T*. *spiralis* infection, and the results indicated that recombinant NC8/TsPPase vaccine was a promising strategy for control of *Trichinella* infection in food animals. This study provides a basis for the development of veterinary anti-*Trichinella* vaccine.
